# Isolation of Multipotent Nestin-Expressing Stem Cells Derived from the Epidermis of Elderly Humans and TAT-VHL Peptide-Mediated Neuronal Differentiation of These Cells

**DOI:** 10.3390/ijms14059604

**Published:** 2013-05-03

**Authors:** Hiroshi Kanno, Atsuhiko Kubo, Tetsuya Yoshizumi, Taro Mikami, Jiro Maegawa

**Affiliations:** 1 Department of Neurosurgery, Yokohama City University, Yokohama 236-0004, Japan; E-Mails: kubstar@msj.biglobe.ne.jp (A.K.); yoshizumi316@tea.ocn.ne.jp (T.Y.); 2 Department of Plastic Surgery, Yokohama City University, Yokohama 236-0004, Japan; E-Mails: zeong3@mac.com (T.M.); maegawaj@med.yokohama-cu.ac.jp (J.M.)

**Keywords:** hair follicle, multipotent stem cells, nestin, neuronal differentiation

## Abstract

A specialized population of cells residing in the hair follicle is quiescent but shows pluripotency for differentiating into epithelial-mesenchymal lineage cells. Therefore, such cells are hoped to be useful as implantable donor cells for regenerative therapy. Recently, it was reported that intracellular delivery of TAT-VHL peptide induces neuronal differentiation of skin-derived precursors. In the present study, we successfully isolated multipotent stem cells derived from the epidermis of elderly humans, characterized these cells as being capable of sphere formation and strong expression of nestin, fibronectin, and CD34 but not of keratin 15, and identified the niche of these cells as being the outer root sheath of the hair follicles. In addition, we showed that TAT-VHL peptide induced their neuronal differentiation *in vitro*, and confirmed by fluorescence immunohistochemistry the neuronal differentiation of such peptide-treated cells implanted into rodent brains. These multipotent nestin-expressing stem cells derived from human epidermis are easily accessible and should be useful as donor cells for neuronal regenerative cell therapy.

## 1. Introduction

The epidermis in humans is a mutilayered epithelium composed of hair follicles, sebaceous glands, and interfollicular epidermis between the orifices of the hair follicles [1]. A specialized nestin-positive cell population residing in the hair follicle has the self-renewing capacity to form spheres and has the potential as multipotent stem cells to differentiate into cells of the not only the epithelial lineage, but also mesenchymal lineage [2]. This specialized cell population is located in the upper, middle, and lower parts of hair follicles in mice [3], whereas it resides in the outer root sheath of hair follicle below the sebaceous glands in humans [2,4]. The cells in this population are also referred to as epidermal neural crest stem cells [5], and are similar to hair follicle dermal papilla stem cells [6], termed skin-derived precursors (SKPs) [7]. It has been suggested that the major source of hair follicle stem cells originate from the hair follicle bulge region [8,9], and nascent blood vessels in the skin were shown to arise from these cells [10]. Recently, non-invasive high-resolution multiphoton tomography was used to observe living cells [11], and by it follicle stem cells were seen to traffic from the hair bulge area towards the dermal papilla [12]. In addition, this population of cells contains both multipotent and monopotent stem cells [13], the both of which are capable of becoming epidermal stem cells [14]; and also SKPs derived from hair follicle stem cells exhibit properties of adult dermal stem cells [15]. The pluripotency of hair follicle stem cells is suggested to be related to epithelial-mesenchymal cross-talk [1]. Markers of cells obtained from human epidermis are keratin 15 and α6-intergrin; whereas CD34 is expressed in the outer root sheath below the bulge region in human hair follicles, though it is highly expressed in the bulge region in mice [16]. Cell markers of human nestin-expressing hair follicle stem cells include nestin and fibronectin but not keratin 15 [16,17]. Previously, nestin-expressing follicle stem cells in adult humans were reported [2,18,19], but hair follicle stem cells obtained from elderly humans over 70 years have never been reported. In addition, both fibronectin and CD34 have never been simultaneously examined as stem cell markers.

Human skin is an attractive source of somatic pluripotent stem cells such as hair follicle stem cells or hair follicle dermal papilla stem cells due to easy access to them. It has been reported that implantation of multipotent nestin-positive hair follicle stem cells promotes the repair of spinal cord and peripheral nerves [8,20–22]. Intractable neuronal diseases such as Parkinson’s disease and numerous other intractable diseases are mostly found in elderly patients. However, little has yet been reported on the isolation of stem cells obtained from the skin of elderly humans. Previously we demonstrated that von Hippel-Lindau tumor suppressor protein (pVHL) induces neuronal differentiation of neuronal progenitor cells [23], and recently, the intracellular delivery of a peptide consisting of amino-acids corresponding to the elongin BC binding site within pVHL into skin-derived precursors, neural stem cells, and bone marrow stromal cells was reported to result in their neuronal differentiation [24–27].

Here we show the isolation of nestin-expressing stem cells from elderly human epidermis and the neuronal differentiation of these cells when VHL-peptide was delivered into them. In addition, we show that these nestin-expressing stem cells simultaneously expressed fibronectin and CD34 but not keratin 15, and identify the niche of these cells as being the outer root sheath of hair follicles. Furthermore, these somatic stem cells implanted into rodent brains were confirmed to be positive for neuronal markers.

## 2. Results

### 2.1. Isolation and Characterization of Multipotent Nestin-Expressing Stem Cells Derived from the Epidermis of Elderly Patients

**Figure 1:**
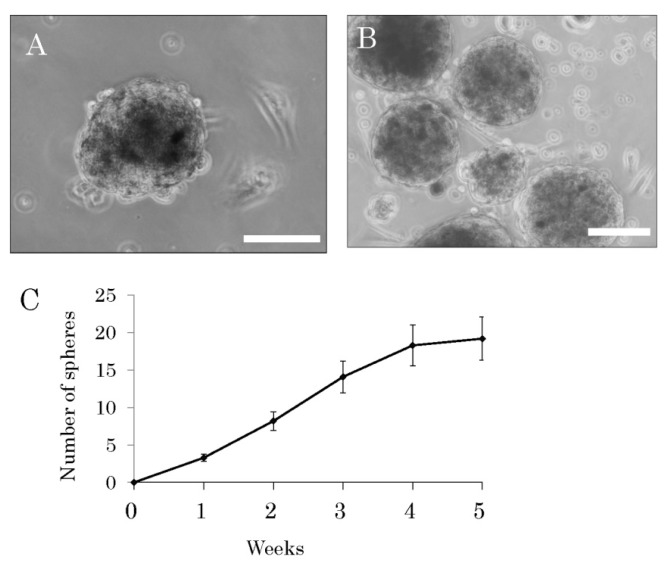
(**A**) One week after the start of a primary culture of cells derived from the epidermis from an elderly human. Sphere formation was recognized; (**B**) Four-week-old primary culture. Numerous floating spheres were observed; (**C**) Results from a sphere-forming assay. After 4 weeks, the rate of sphere formation became slow. Scale bars = 50 μm.

**Figure 2:**
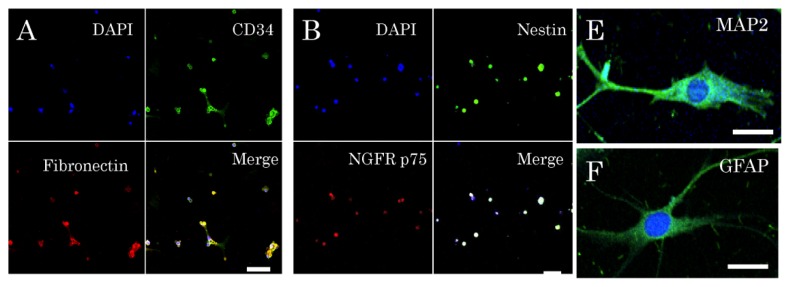

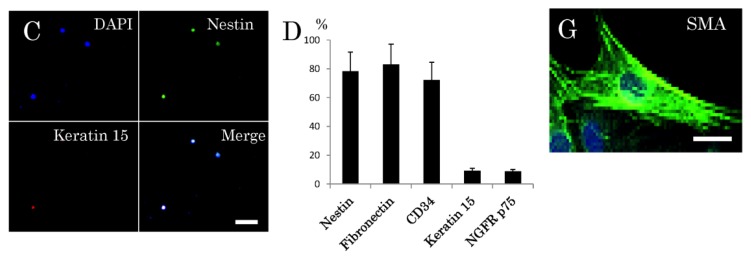
Fluorescence immunocytochemistry of the cultured stem cells after dissociation of the spheres into single cells. The spheres at 5 weeks in primary culture were dissociated by pipetting. (**A**) Triple fluorescence immunocytochemistry for expressions of CD34 (green) and fibronectin (red) and visualization of nuclei (blue); (**B**) Triple fluorescence immunocytochemistry for nestin (green), NGFR p75 (red), and nuclei (blue); (**C**) Triple fluorescence immunocytochemistry for nestin (green), keratin 15 (red), and nuclei (blue); (**D**) Percentages of the cells showing expression of the five proteins examined. High expressions of nestin, fibronectin, and CD34 were found, whereas those of keratin 15 and NGFR p75 were low. DAPI, nuclear marker; Merge, all three images superimposed; (**E**–**G**) Fluorescence immunocytochemical study on differentiation of stem cells into neurons expressing microtubule-associated protein (MAP)-2 (green, **E**), astrocytes expressing glial fibrillary acidic protein (GFAP; green, **F**), and smooth muscle cells expressing smooth muscle actin (SMA; green, **G**), with the nucleus in blue. Scale bars = 50 μm.

Facial skin samples were obtained from 16 elderly patients having a complaint of ptosis, which is symptomatically the downward positioning or drooping of the upper eye-lid that occurs with aging, prior to plastic surgery. The patients (6 males and 10 females) ranged in age from 55 to 86 years with a mean age of 69.1 ± 9.5 years, and seven patients were over 70 years of age (the oldest patient, 86 years). Samples of the surplus skin removed at surgery for ptosis were used for this study. The epidermis, rich in hair follicles, was peeled off from the dermis after dispase treatment. Then, the epidermis was cut into small fragments, digested with trypsin, and filtered through a 40-μm-pore cell strainer. After having been dissociated into single cells, the epidermal cells were cultured in medium consisting of DMEM/F12 containing B27 supplement, basis fibroblast growth factor, and epidermal growth factor, but no serum. The cells were observed under a phase-contrast microscope every 3–4 days starting one day after the establishment of the primary cultures. The stem cells among them started to form spheres one week after the start of the primary cultures, and these spheres grew little by little (Figure 1A). By 4 weeks numerous spheres were found floating in the culture medium (Figure 1B). Although the sphere formation, being a reflection of self-renewal capacity, was recognized as having been due to these stem cells, the cell growth became slower after the fourth week (Figure 1C). Among the cells dissociated from spheres in six-weeks-primary cultures, 78.3% ± 12.1% of them were positive for nestin; 83.1% ± 11.5%, positive for fibronectin; and 72.3% ± 10.3%, positive for CD34. In contrast, only 9.3% ± 1.4% of them showed expression of keratin 15; and only 8.7% ± 1.2%, that of NGFP p75 (Figure 2D). One week after dissociation of these sphere-forming cells, the single cells cultured in medium consisting of DMEM/F12 medium containing 1% fetal calf serum without growth factors or supplement differentiated into neuron-marker (microtubule-associated protein, MAP)-2-positive cells (Figure 2E), astrocyte-marker (glial fibrillary acidic protein, GFAP)-positive cells (Figure 2F) or smooth muscle marker (smooth muscle actin, SMA) -positive cells (Figure 2G).

### 2.2. Niche of Multipotent Nestin-Expressing Stem Cells Derived from Epidermis of Elderly Humans

**Figure 3:**
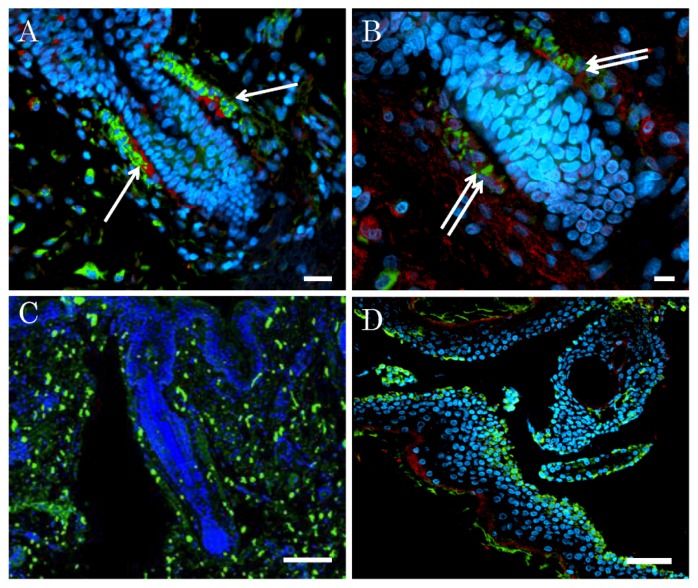
Fluorescence immunohistochemistry for skin, including epidermis and dermis, and epidermis only. (**A**) Triple fluorescence immunohistochemistry for expressions of nestin (green) and CD34 (red), and visualization of nuclei (blue) in the skin. Both nestin and CD34-co-expressing cells (arrow) are shown at the outer root sheath of hair follicles; (**B**) Triple fluorescence immunohistochemistry for expressions of nestin (green) and fibronectin (red), and visualization of nuclei (blue) in the skin. Both nestin and fibronectin-co-expressing cells (double arrows) are seen at the outer root sheath of hair follicles; (**C**) Double fluorescence immunohistochemistry for expression of keratin 15 (green) and visualization of nuclei (blue). Keratin 15 was identified in the hair follicle and keratinocyte layer; (**D**) Triple fluorescence immunohistochemistry of epidermis alone for nestin (green) and keratin 15 (red) and visualization of nuclei (blue). A nestin-expressing cell population was identified in the hair follicles. Keratin 15 was expressed in the outer layer. Scale bars = 50 μm.

In our pathological analysis of elderly human whole skin including both epidermis and dermis, the niche of nestin-expressing stem cells derived from the epidermis was identified to be the outer root sheath region of the hair follicles, which cells showed high triple expressions of nestin, fibronectin, and CD34 (Figure 3A,B). On the other hand, high expression of keratin 15 was detected in both the follicle and the interfollicle regions (Figure 3C). Thus the location of the triple (nestin, fibronectin, and CD34)-expressing cells was different from that of the keratin 15-expressing ones. Except for the bulge region, some of the dermal tissue beneath the epidermis contained a population of cells immunopositive for these triple markers. In addition, in the pathological analysis of the epidermis only (Figure 3D), the sites of nestin and keratin 15 were completely different. These results suggested that the niche of nestin-expressing cells was the outer root sheath of the hair follicles.

### 2.3. Neuronal Differentiation of Stem Cells after Intracellular Delivery of TAT-VHL Peptide

**Figure 4:**
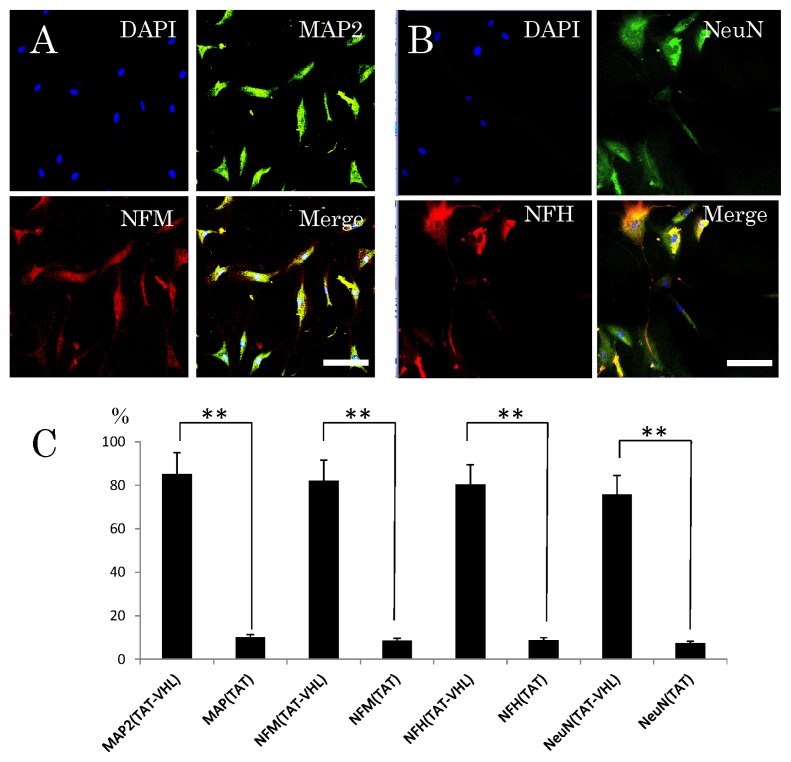
Fluorescence immunocytochemistry of the cultured stem cells after intracellular delivery of TAT-VHL peptide. (**A**) Triple fluorescence immunocytochemistry for expressions of MAP2 (green) and neurofilament-M (NFM, red) and visualization of nuclei (blue); (**B**) Triple fluorescence immunocytochemistry for NeuN (green) and neurofilament-H (NFH, red) and visualization of nuclei (blue); (**C**) Percentages of the TAT-VHL peptide- or TAT peptide-containing cells expressing MAP2, NFM, NFH or NeuN. The TAT-VHL peptide cells showed significantly higher expression of all 4 proteins (** *p* < 0.001). Scale bars = 30 μm.

Neuronal differentiation of the stem cells was examined after the intracellular delivery of TAT-VHL peptide, which has been reported to have the ability to induce neuronal differentiation [25–27]. For the control, the neuronal differentiation of cells treated with TAT-peptide was also examined. At first, an *in vitro* study was done. Two days after the intracellular delivery of TAT-VHL peptide at a 1-μM concentration in DMEM/F12 medium without growth or neurotrophic factors, phase-contrast microscopy and an immunocytochemical study were performed. Observation by phase-contrast microscopy showed that most cells after transfer of the TAT-VHL peptide into them grew neurite-like cellular processes (78.3% ± 12.5%), whereas the control cells receiving TAT peptide extended significantly fewer of these processes (18.6% ± 7.3%). In the immunocytochemical *in vitro* study, the TAT-VHL peptide-containing stem cells derived from the epidermis showed high expression of various neuronal markers (MAP2, Neurofilament-M, Neurofiament-H, and Tuj-1), whereas those containing the control TAT peptide showed low expression of these markers (Figure 4A–C).

### 2.4. Implantation of Mutipotent Nestin-Expressing Stem Cells into the Rodent Brain

**Figure 5:**
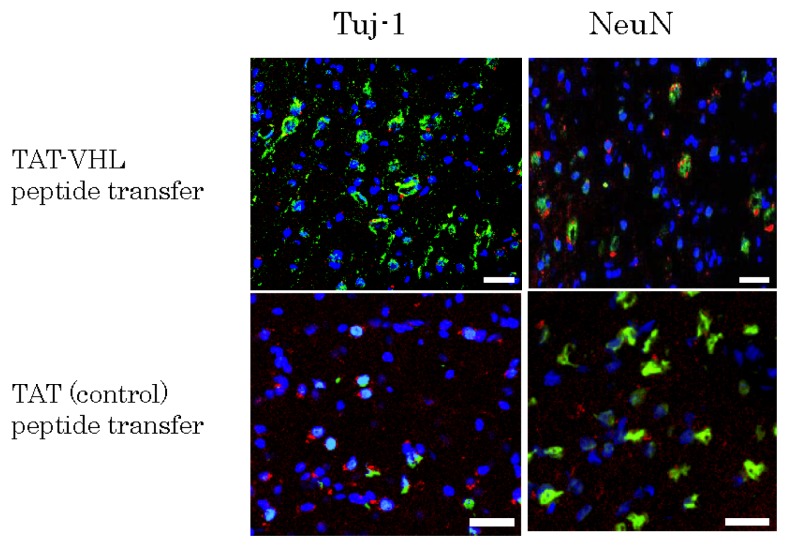
Fluorescence immunohistochemistry for rodent brain tissues implanted with peptide-transferred nestin-expressing stem cells pre-stained with red-fluorescent PKH26-PCL. Confocal immunohistochemical images showed expression of neuronal marker Tuj-1 (green) or NeuN (green). The nuclei were stained with DAPI (blue). PKH-pre-stained TAT-VHL peptide-containing cells showed significantly more expression of Tuj-1 and NeuN than did the pre-stained TAT peptide-containing ones (*p* < 0.01). Scale bar = 20 μm.

TAT-VHL peptide-containing epidermal stem cells and control ones containing TAT peptide were pre-stained with red fluorescent PKH26, and separately implanted into Wistar rat brains. Three weeks later, after perfusion/fixation, the brains were frozen with liquid nitrogen and sectioned at 10 μm. Then, using anti-Tuj-1 and anti-NeuN antibodies, we performed an immunohistochemical study on these sections. Nuclei were stained with DAPI. The number of surviving implanted cells (red fluorescent PKH-pre-stained cells) among the TAT-VHL peptide-containing implanted cells (20.4% ± 2.7%) was significantly greater than that of the TAT peptide-containing ones (6.6% ± 0.8%, *p* < 0.01). In addition, PKH-pre-stained cells expressing Tuj-1 represented 38.8% ± 3.5% of the TAT-VHL peptide-containing cell population, which percentage was significantly greater (*p* < 0.01) than the 9.3% ± 1.5% found for the TAT peptide-containing one (Figure 5).

## 3. Discussion

In this report, we demonstrated the isolation of mutipotent nestin-expressing stem cells derived from the epidermis of facial skin obtained from elderly humans (mean 69.1 years of age), and showed the neuronal differentiation of these cells when the TAT-VHL peptide was intracellularly delivered into them. The isolated cells showed sphere-forming ability and high expression of triple markers (fibronectin, nestin, and CD34), but very low expression of keratin 15 and NGFR p75. These findings are compatible with those on human hair follicle stem cells [2]. In addition, the results of our experiment to identify the niche of the stem cells suggested that the isolated nestin-positive stem cells originated from the outer sheath root of hair follicles. Murine multipotent nestin-expressing stem cells, derived from either the hair follicle bulge area or dermal papilla possess sphere-forming capacity [8]. Our isolated multipotent nestin-positive cells also showed sphere-forming ability, which reflects self-renewing capacity [28], and was also found in the case of skin-derived neural crest stem cells [29] or skin-derived precursors [7]. Our isolated stem cells included the cells obtained from seven patients over 70 years of age (the oldest patient, 86 years old), and so our results indicate that skin-derived nestin-expressing follicle stem cells could be isolated even from patients over 70 years of age. In addition, our data clearly identified the niche of these stem cells; whereas most previous studies did not fully elucidated the stem cell niche and also never examined the expression of both fibronectin and CD34 simultaneously.

Multipotent nestin-expressing stem cells are promising as donor cells for the treatment of intractable neuronal diseases [20,30]. However, if these cells without neuronal differentiation are implanted, they scarcely survive or differentiate to functional neuronal cells, similar to the case of other stem cells. Therefore, before implantation for cell therapy of intractable neuronal diseases, such cells would be required to differentiate into neuronal cells. Our data showed that the stem cells treated *in vitro* with TAT-VHL peptide and implanted into rat brains survived and differentiated into neuronal marker-positive cells, even though the implantation was into another mammalian species, probably because the immune system in the central nervous system was not fully functional by the end of the short period of observation and because VHL protein contributes to the survival of implanted cells [31–33]. The elongin BC-binding site within the VHL protein is a domain that induces the neuronal differentiation of various stem cells [24–27], and so the intracellular delivery of the amino-acid sequence corresponding to this binding site would have brought about the neuronal differentiation of these somatic stem cells. In previous studies, it was shown that the intracellular delivery of TAT-VHL peptide induces not only *in vitro* neuronal differentiation of somatic stem cells but also *in vivo* neuronal differentiation and recovery from neuronal symptoms in neuronal disease models [25–27]. In addition, it was recently demonstrated that implantation of skin-derived precursors into which TAT-VHL peptide had been intracellularly delivered not only improved symptoms of Parkinson’s model rats but also resulted in the secretion of dopamine in the rodent striatum [34]. Recently it was reported that implantation of human follicle pluripotent stem cells promotes regeneration from peripheral-nerve injury in nude mice [20,22]. We here showed that multipotent nestin-expressing stem cells isolated from the epidermis obtained from elderly humans differentiated into neuronal-marker positive cells even though xenografted into the brains of non-immune-deficient rats. Human leukocyte antigen (HLA)-type adaptable allograft implantation of nestin-expressing follicle stem cells into the central nervous system (CNS) might be tolerable without rejection and contribute to the promotion of CNS regeneration. Intractable neuronal diseases such as Parkinson’s disease develop more frequently in elderly patients. Therefore, it is significant that multipotent stem cells derived from the epidermis can be obtained from elderly humans as well as from younger individuals. Basically, multipotent somatic stem cells do not transform into cancer cells, whereas iPS cells do; and the problem of malignant transformation of these cells has still not be solved. In practical use for human neuronal regeneration, multipotent somatic stem cells are considered to be superior to iPS cells. The mechanism of the neuronal differentiation of stem cells in response to the intracellular delivery of TAT-VHL peptide is not fully elucidated. It has been suggested that the binding of TAT-VHL peptide to elongin BC is competitive with the binding of elongin BC to elongin A, which is a critical reaction in messenger RNA elongation [35]. In addition, it was recently suggested that the VHL protein inhibits Stat3, which plays a decisive role in astroglial differentiation [28]. This phenomenon might be related to the neuronal differentiation in response to the intracellular delivery of TAT-VHL peptide. The epidermis is an easily accessible source. Recently, truly pluripotent SSEA-3-positive MUSE cells were isolated from a skin fibroblast population by use of a cell sorter [36]. Although the population of somatic stem cells derived from the epidermis might contain MUSE cells, it may not be necessary to re-isolate MUSE cells from among such somatic stem cells since those stem cells themselves can differentiate into cells of the epithelial lineage in various organs. The neuronal differentiation method using TAT-VHL peptide is very simple because only the TAT-VHL peptide needs to be added to basic medium lacking serum, and it is also rapid compared with previously reported methods using various neurotrophic factors and other agents. Thus, we recommend the use of TAT-VHL for the neuronal differentiation of multipotent somatic stem cells.

## 4. Experimental Section

### 4.1. Cell Culture

Samples of human skin were sterilized with 70% ethanol for 1 min. Then, the skin was treated with dispase (concentration 1000 PU/mL) for 24 h at room temperature. Next, the epidermis was peeled off from the dermis, cut into small fragments with scissors, and digested with 0.1% trypsin for 30 min at 37 °C. Thereafter, the cells were filtered through a cell strainer (40-μm-diameter holes) and subsequently cultured in medium containing epidermal growth factor (20 ng/mL), basic fibroblast growth factor (40 ng/mL), and 2% B27 supplement (Gbico-BRL, Grand Island, NY, USA) in DMEM/F12 (1:1; Gibco-BRL) in a 5% CO_2_ incubator. The cells formed numerous spheres at 2–3 weeks after the start of the primary culture. Once the primary culture was 3–4 weeks old, the spheres were dissociated by pipetting; and the cells were subcultured every two weeks. For experiments, cells at the first or second subculture were used.

### 4.2. Identification of Niche of Multipotent Nestin-Expressing Stem Cells Derived from Human Epidermis

For identification of the niche of pluripotent sphere-forming stem cells derived from the epidermis, a part of the cut skin, which had been fixed with Mildform (Wako, Tokyo, Japan), was embedded in paraffin, sectioned at a 10-μm thickness, and immunostained with anti-nestin antibody (BD Bioscience PharMingen, San Diego, CA, USA), anti-fibronectin antibody (Sigma, St. Louis, MO, USA), anti-CD34 antibody (Sigma) and anti-keratin 15 antibody (Acris Antibodies GmbH, Herford, Germany). As secondary antibodies, FITC-conjugated anti-mouse IgG monoclonal antibody (Sigma) and TRIC-conjugated anti-rabbit IgG polyclonal antibody were used. Finally, the nuclei were stained with DAPI (Molecular Probes, Eugene, OR, USA). Observation was performed by using a confocal fluorescence microscope (FV300, Olympus, Tokyo, Japan).

### 4.3. Immunocytochemical Characterization of Multipotent Stem Cells

After dissociation of the sphere-forming cells, characterization of the stem cells was performed immunocytochemically by using anti-nestin antibody, rabbit anti-fibronectin antibody (1:200; Sigma), anti-CD34 antibody, anti-NGFR p75 (Sigma), and anti-kerain 15 antibody (Acris Antibodies GmbH). For examination of stem cell differentiation into certain cell types, ant-glial fibrillary acidic protein (GFAP) antibody (1:300; Dako, Glostrup, Denmark), anti-smooth muscle actin (SMA) antibody (1:200; Sigma), and anti-microtubule-associated protein (MAP)-2 (1:300; Sigma) were used. Immunoreactive cells were visualized by using either FITC-conjugated goat anti-mouse IgG (1:200; Sigma) or rhodamine-conjugated goat anti-rabbit IgG (1:150; Sigma) as secondary antibodies. To assess the frequency of different cell types in a given culture, we counted the number of cells immunopositive with a given antibody in 10 to 15 random non-overlapping visual fields (50–200 cells per field) in each experiment. At least three experiments were performed per condition. The degree of positivity was expressed as the ratio of immuno-positive cells to the total number of nuclei stained with DAP1.

### 4.4. Sphere-Forming Assay

The sphere formation assay was performed as follows [28]: Sphere-forming stem cells were dissociated into single cells by pipetting them continuously for 10 min. Then, after confirmation of their single-cell status and dilution up to 5 cells/mL, 200 μL of the cell suspension was placed into each well of a 96-well plate (mean of 1 cell per well). Then, spheres ≥50 μm in diameter in 1 plate were counted under observation by phase-contrast microscopy at 3 weeks after placement of the cells.

### 4.5. Induction of Neuronal Differentiation with TAT-VHL Peptide

By using the Fmoc (9-fluorenylmethyloxycarbonyl)-solid-phase method described previously [24], we chemically synthesized a peptide corresponding to the 157–171 amino-acid sequence of pVHL. The synthesized peptide was linked with the protein transduction domain (PTD) of the HIV-TAT protein (TAT-VHL peptide), thereby facilitating peptide entry into the cells. A control peptide composed of only the PTD of the TAT protein (TAT-peptide) was also synthesized. The sequences of these synthesized peptides were NH_2_-YGRKKRRQRRRDTLKERCLQVVRSLVK-COOH for the TAT-VHL peptide and NH_2_-YGRKKRRQRRRD-COOH for the TAT one. The isolated stem cells were incubated in DMEM/F12, and 1 μM TAT-VHL peptide or TAT peptide was delivered into the dissociated stem cells for neuronal differentiation. At first, an *in vitro* immunocytochemical study was performed. Three days after intracellular delivery of the TAT-VHL peptide in DMEM/F12 medium without growth or neurotrophic factors, the cells were observed under the phase-contrast microscope, and an immunocytochemical study was performed by using the following antibodies: anti-MAP2 antibody (1:200; Sigma), anti-tyrosine hydroxylase antibody (TH; 1:200; Merk Millipore, Billerica, MA, USA), and anti-neurofilament 200 antibody (1:200; Sigma). Immunoreactive cells were visualized by using either FITC-conjugated goat anti-mouse IgG (1:200; Sigma) or rhodamine-conjugated goat anti-rabbit IgG (1:150; Sigma) as secondary antibodies. In addition, DAPI (Molecular Probes) was used for counterstaining nuclei.

### 4.6. Implantation of Multipotent Stem Cells Derived from Epidermis

One hour after the TAT-VHL peptide had been transferred into the stem cells, the cells were stained with red-fluorescent PKH26PCL (Sigma), and implanted into the brains of eight-week-old Wistar rats (Charles River, Yokohama, Japan). Three weeks after the implantation, the rats were anesthetized with Nembutal (200 mg/kg body weight) and perfused with periodate-lysine-paraformaldehyde solution. Their brains were subsequently dissected and postfixed in the same fixative for 2 h, cryopreserved in 30% sucrose for 12 h, and then embedded in Tissue Tek OCT compound (Sakura, Tokyo, Japan). Cryostat coronal sections of 14-μm thickness were prepared and used for immunohistochemistry. For immunostaining, sections were incubated with primary antibody, *i.e.*, anti-NeuN antibody (1:200; Merk Millipore, Billerica, MA, USA) or anti-Tuj-1 antibody (1:200; R&D Systems, Minneapolis, MN, USA) for 1 h at room temperature. Immunoreactive cells were visualized by using either FITC-conjugated goat anti-mouse IgG (1:200; Sigma) or rhodamine-conjugated goat anti-rabbit IgG (1:150; Sigma) as secondary antibodies. In addition, DAPI was used for counterstaining nuclei.

### 4.7. Statistics

Results were expressed as the mean ± standard deviation. For comparisons between values for groups, the Scheff test after the ANOVA test was used, with probabilities of less than 0.05 being considered significant (Statcel version 5.0/7.0, California, CA, USA).

### 4.8. Ethical Approval

This study was approved by the Ethics Committee of Yokohama City University in 2009, and the informed consent for this study was obtained from all of the patients.

## 5. Conclusions

In conclusion, we showed the isolation of multipotent nestin-expressing stem cells from the epidermis of facial skin of elderly humans and characterized these cells as being capable of sphere formation and strong expression of nestin, fibronectin, and CD34 but not of keratin 15. In addition, we identified the niche of these stem cells as being the outer root sheath of hair follicles and showed neuronal differentiation of these cells both *in vitro* and *in vivo* when VHL-peptide had been delivered into them. These multipotent stem cells derived from human epidermis are easily accessible and should be useful as donor cells for neuronal regenerative cell therapy.
